# Enhanced UV-Reflection Facilitated a Shift in the Pollination System of the Red Poppy, *Papaver rhoeas* (Papaveraceae)

**DOI:** 10.3390/plants9080927

**Published:** 2020-07-22

**Authors:** Jaime Martínez-Harms, Ravit Hadar, Natalia Márquez, Randolf Menzel, Avi Shmida, Doekele G. Stavenga, Misha Vorobyev

**Affiliations:** 1 Institut für Biologie-Neurobiologie, Freie Universität Berlin, Königin-Luise Str. 1–3, 14195 Berlin, Germany; ravit.hadar@charite.de (R.H.); nmarquez@zedat.fu-berlin.de (N.M.); menzel@zedat.fu-berlin.de (R.M.); 2 INIA La Cruz, Instituto de Investigaciones Agropecuarias, Chorrillos 86, La Cruz 2280454, Chile; 3 Department of Ecology, Evolution & Behaviour, Center for the Study of Rationality, The Hebrew University, Jerusalem 91904, Israel; shmida@math.huji.ac.il; 4 Zernike Institute for Advanced Materials, University of Groningen, Nijenborgh 4, NL-9747 AG Groningen, The Netherlands; d.g.stavenga@rug.nl; 5 School of Optometry and Vision Science, University of Auckland, Private Bag 92019, Auckland 1142, New Zealand; m.vorobyev@auckland.ac.nz

**Keywords:** flower color, pollination systems, color vision, biological invasions

## Abstract

Evolutionary change is considered a major factor influencing the invasion of new habitats by plants. Yet, evidence on how such modifications promote range expansion remains rather limited. Here we investigated flower color modifications in the red poppy, *Papaver rhoeas* (Papaveraceae), as a result of its introduction into Central Europe and the impact of those modifications on its interactions with pollinators. We found that while flowers of Eastern Mediterranean poppies reflect exclusively in the red part of the spectrum, those of Central European poppies reflect both red and ultraviolet (UV) light. This change coincides with a shift from pollination by glaphyrid beetles (Glaphyridae) to bees. Glaphyrids have red-sensitive photoreceptors that are absent in bees, which therefore will not be attracted by colors of exclusively red-reflecting flowers. However, UV-reflecting flowers are easily detectable by bees, as revealed by visual modeling. In the North Mediterranean, flowers with low and high UV reflectance occur sympatrically. We hypothesize that Central European populations of *P. rhoeas* were initially polymorphic with respect to their flower color and that UV reflection drove a shift in the pollination system of *P. rhoeas* that facilitated its spread across Europe.

## 1. Introduction

A major environmental perturbation induced by dispersal of plants to new habitats is the decoupling from their native pollinators. The lack of pollinators can induce pollen limitation, constraining the spread of plants into new environments [1,2]. Within this context, and given that species with generalized pollination systems are more likely to find pollinators in new environments, it has been hypothesized that pollination specialization would represent a barrier for the colonization of new habitats by plants [3]. Previous reports have shown that chronic outcross pollen limitation can lead to the evolution of mechanisms of self-pollination [4,5]. Evolutionary changes of floral traits could also help ensure the reproduction of plants exposed to sudden changes in pollinator environment [6]. Attributes such as flower color, odor, and shape play an important role in attracting pollinators. Modifications of such traits could contribute to maintaining outcrossing by facilitating interactions with novel pollinators [6].

The red poppy, *Papaver rhoeas*, is an iconic plant whose cultural relevance can be traced back several millennia. Its ornamental, pharmacological, and edible value was already recognized as early as the 18th dynasty in Egypt (1504–1450 BC) [7]. Taxonomic evidence suggests that *P. rhoeas* originated in the East Mediterranean [8], from where it presumably disseminated as an agricultural weed together with the expansion of farming practices along the coast of the Mediterranean around 8000 years ago [9,10,11]. Fossil evidence indicates that the red poppy spread into Central Europe approximately 5000 years ago [10,11,12]. This spread decoupled the red poppy from its native pollinators, inducing a shift from a rather specialized form of pollination by glaphyrid beetles (Glaphyridae: Coleoptera) to a form of pollination that includes social and solitary bees as main pollinators, as well as flies and beetles [13,14,15,16]. Despite being self-incompatible [17,18,19] and therefore dependent on pollinators for reproduction, *P. rhoeas* successfully established self-perpetuating populations (i.e., it became naturalized) in human-made habitats across Europe [8,10,11,12].

Glaphyrids and bees differ substantially with respect to their receptor-based color vision. The glaphyrid beetle *Pygopleurus israelitus* has receptors maximally sensitive to UV, green, and red [20], while most bees have trichromatic color vision based on receptors maximally sensitive to UV, blue, and green [21]. These severe differences in spectral photoreceptors will have important implications in the way beetles and bees perceive red flowers [20,22,23], and presumably this should affect their association with *P. rhoeas*. Considering that color is a major attractant for glaphyrids and bees, we asked whether the geographical spread of *P. rhoeas* caused modifications of the color of its flowers. To answer this question, we evaluated the spectral reflectance properties of flowers of *P. rhoeas* in populations in the East and North Mediterranean and Central Europe and modeled flower color appearance, taking into account the spectral sensitivities of the photoreceptors of the glaphyrid beetle, *Pygopleurus israelitus* (Glaphyridae), and the most common bee pollinator, the honeybee *Apis mellifera*.

## 2. Results

**Figure 1:**
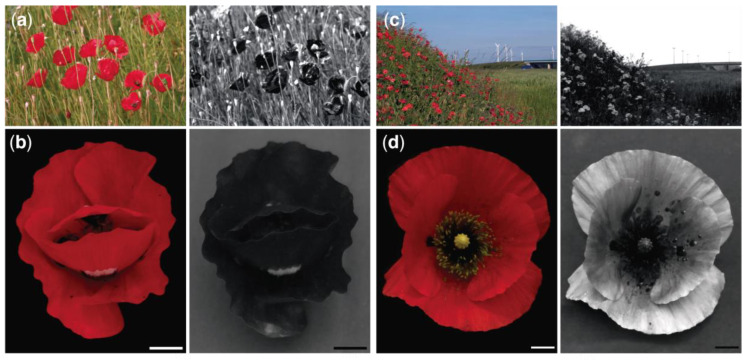
RGB and UV images of flowers of *P. rhoeas*. (**a**) A field containing flowers of *P. rhoeas* in the East Mediterranean. (**b**) A *P. rhoeas* flower collected in the East Mediterranean. (**c**) A field with *P. rhoeas* flowers in Central Europe. (**d**) A *P. rhoeas* flower collected in Central Europe. Scale bars: (b,d) 1 cm.

**Figure 2:**
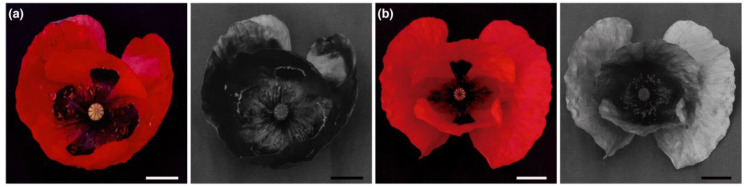
RGB and UV images of flowers of *P. rhoeas* collected in the North Mediterranean. (**a**) A *P. rhoeas* flower that reflects negligibly in UV. (**b**) A *P. rhoeas* flower with appreciable UV reflectance. Scale bars: 1 cm.

For human eyes, the East Mediterranean *P. rhoeas* is indistinguishable from its European counterpart (Figure 1a–d). However, under ultraviolet (UV) light, the flowers of East Mediterranean and European *P. rhoeas* appear strikingly different. In UV images of poppy fields, the flowers of East Mediterranean *P. rhoeas* look like dark spots, while blossoms of the European poppy create bright spots (Figure 1a,c). These images demonstrate that the UV reflectance of the East Mediterranean *P. rhoeas* is lower than that of the green vegetation background, whereas European *P. rhoeas* reflects UV more strongly than the green vegetation background. UV images of individual flowers show that the East Mediterranean flowers reflect UV negligibly while the UV reflectance of European flowers is appreciable, except for the central area with stamens (Figure 1b,d). Images taken in the North Mediterranean revealed flowers with negligible or appreciable UV reflectance occurring sympatrically in this region (Figure 2a,b).

**Figure 3:**
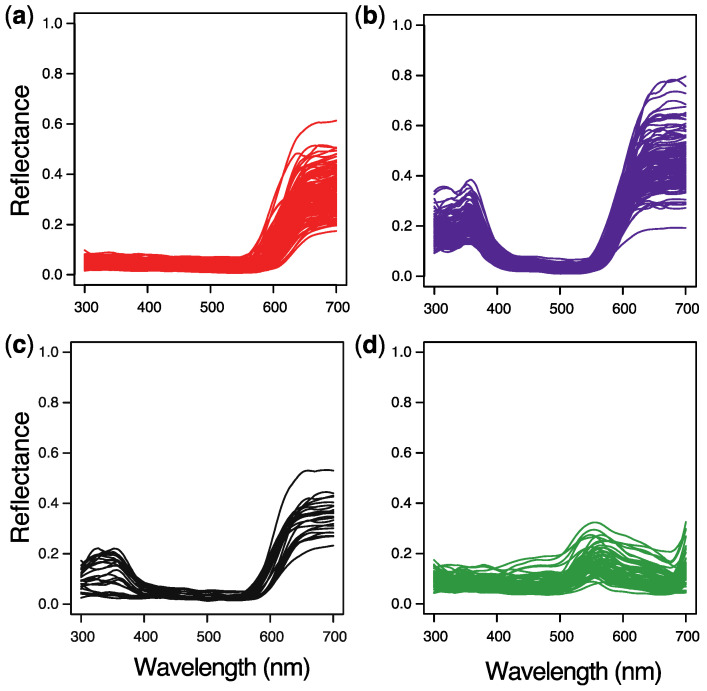
Reflectance spectra of flowers and foliage of *P. rhoeas* collected in the field and measured with a spectrometer setup with two optical fibers. (**a**) Reflectance spectra of flowers collected in the East Mediterranean (n = 124). (**b**) Reflectance spectra of flowers collected in Central Europe (n = 116). (**c**) Reflectance spectra of flowers collected in the North Mediterranean (n = 22). (**d**) Reflectance spectra of green foliage (n = 77).

The reflectance spectra of both East and North Mediterranean as well as European red poppies showed a high reflectance at wavelengths >590 nm, which accounts for their bright red color (Figure 3a–c). However, whereas the East Mediterranean flowers (n = 124) had a low UV reflectance (Figure 3a), accounting for their dark appearance in UV images (Figure 1a,b), the spectra of all investigated European flowers (n = 116) showed an enhanced UV reflectance, between 300 and 400 nm (Figure 3b), corresponding to their bright appearance in UV images (Figure 1c,d). Interestingly, measurements taken in the North Mediterranean revealed flowers (n = 22) with widely varying UV-reflectance levels, all occurring sympatrically in this region, i.e., with low and intermediate as well as enhanced UV reflectance (Figure 3c). Our results are consistent with studies that reported the absence of UV reflectance in *P. rhoeas* flowers from Israel [14] and the presence of UV reflectance in European *P. rhoeas* flowers [24,25,26,27,28].

Our results demonstrate that European and North and East Mediterranean populations of *P. rhoeas* have differentiated with respect to their flower color. We note here that we identified only a single UV-reflecting flower in UV photographs of a poppy field in Israel. This indicates that UV-reflecting flowers may not be totally absent in the East Mediterranean, occurring in low numbers during the peak of *P. rhoeas*’ flowering. Data collected in the North Mediterranean reveal a transition zone where flowers with low and high UV reflectance occur sympatrically. We hypothesize that Central European populations of *P. rhoeas* were initially polymorphic with respect to their UV reflectance, as still holds for the North Mediterranean populations, and that the low-UV-reflectance flowers were lost in this region. Alternatively, Central European populations of *P. rhoeas* could have been founded by individuals having UV-reflecting flowers only.

In the East Mediterranean, populations of *P. rhoeas* are mainly pollinated by beetles from the family Glaphyridae [14], while in Central Europe, *P. rhoeas* is mainly pollinated by bees [13,15,16]. Glaphyrid beetles and hymenopterans differ substantially with respect to the spectral sensitivity of their photoreceptors. Whereas the glaphyrid beetle *Pygopleurus israelitus* has receptors maximally sensitive to UV, green, and red light [20], most hymenopterans have receptors maximally sensitive to UV, blue, and green [21] (Figure 3a,b). We investigated whether glaphyrid beetles and bees can detect and discriminate flowers of *P. rhoeas* with different UV reflectance by modeling the flowers’ color appearance using the different spectral sensitivities of the photoreceptors of these pollinators and two chromaticity diagrams, i.e., the receptor noise-limited (RNL) color-opponent model and the Maxwell triangle.

**Figure 4:**
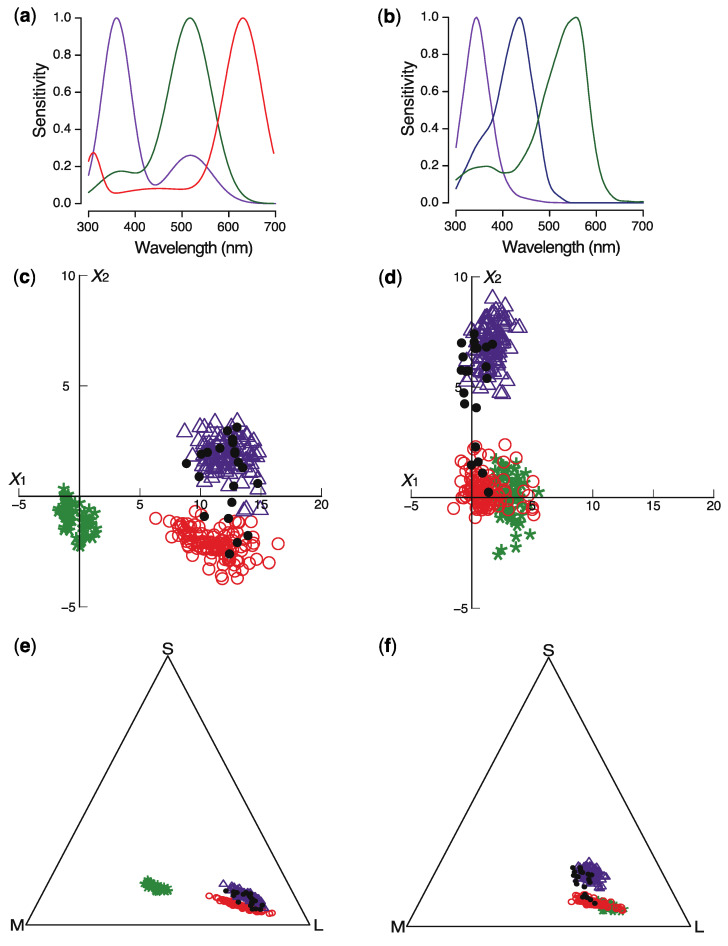
Modeling of flower color appearance for pollinators. (**a**) Spectral sensitivities of UV-, green-, and red-sensitive photoreceptors of *Pygopleurus israelitus*. (**b**) Spectral sensitivities of UV-, blue-, and green-sensitive photoreceptors of honeybees. (**c**) Colors of *P. rhoeas* represented in the chromaticity diagram of *P. israelitus* according to the receptor noise-limited (RNL) color-opponent model. (**d**) Colors of *P. rhoeas* represented in the chromaticity diagram of the honeybee according to the RNL color-opponent model. (**e**) Colors of *P. rhoeas* represented in the chromaticity diagram of *P. israelitus* according to the Maxwell triangle. (**f**) Colors of *P. rhoeas* represented in the chromaticity diagram of the honeybee according to the Maxwell triangle. The red circles represent the colors of negligibly UV-reflecting flowers from the East Mediterranean. The violet triangles represent the colors of flowers from Central Europe that reflect well in the UV. The black dots represent the colors of flowers collected in the North Mediterranean. The green asterisks represent the colors of green foliage.

We plotted loci of Mediterranean and Central European *P. rhoeas* (n = 262) and of green foliage (n = 77) in the chromaticity diagrams of the glaphyrid beetle *Pygopleurus israelitus* and the most common bee pollinator, the honeybee *Apis mellifera* (Figure 4c–f). For the glaphyrid beetle, both chromaticity diagrams show that colors of *P. rhoeas* flowers with low and high UV reflectance are clearly different from each other as well as from green foliage (Figure 4c,e). For a bee, the colors of red poppies with low UV reflectance and green foliage occupy overlapping loci in both chromaticity diagrams, while the colors of UV-reflecting flowers and green foliage are distinctly different (Figure 4d,f). We also calculated the colors of flowers collected in the North Mediterranean. The flowers with low or high UV reflectance occupied loci overlapping with flowers from the East Mediterranean or Central Europe (Figure 4c–f). Not surprisingly, the loci of flowers with intermediate levels of UV reflectance occupy loci located between these two groups (Figure 4c–f).

In line with previous work showing that trichromatic bees perceive pure red flowers through achromatic mechanisms [22,29], our calculations show that bees cannot discriminate colors of red poppies with low UV reflectance from a green background on the basis of chromatic cues, but they can easily discriminate colors of UV-reflecting poppies from green foliage. This indicates that populations of European and East Mediterranean *P. rhoeas* have differentiated with respect to their flower appearance to available pollinators. Additionally, the modeling indicates that in the North Mediterranean, pollinators encounter flowers of *P. rhoeas* with various color appearances.

A possible caution raised to the calculations is the spectral sensitivity of the beetle’s UV receptor, which has a distinct band in the green wavelength range (Figure 4a). The possibility that this band is an artefact due to the measurement procedure cannot be fully ruled out [20]. We therefore recalculated the beetle’s chromaticity diagram assuming a UV receptor with a single sensitivity band in the UV. As the resulting loci were virtually identical to those shown in Figure 4c, our conclusions on the discriminatory capacities of the beetle remain the same.

## 3. Discussion

Evolutionary change has long been recognized as an important process in the invasion of new habitats by plants [3]. Yet, evidence for a direct role of such modifications in promoting range expansion remains rather limited [30,31,32]. Within this context, it has been argued that evolutionary modifications contributing to ensure reproduction in novel environments would influence the rate and patterns of geographical range expansion [6,33]. Specialization to restricted groups of pollinators has been hypothesized to represent a barrier to biological invasions [3,34]. The evidence presented here shows that modifications in flower attractive traits can drive shifts in pollination systems, allowing plants to overcome these barriers. Along its native range, the red poppy presents a rather specialized form of pollination by glaphyrid beetles [14,20], from which it was decoupled as it was introduced into Central Europe [8,10,11,12]. Since then, Central European populations of *P. rhoeas*, by differentiating with respect to the UV reflectance of their flowers, could successfully attract a different guild of pollinators.

The low UV reflectance of the Mediterranean poppies is presumably caused by UV-absorbing pigments, the concentration of which, in Central European poppies, is severely diminished [35]. Pigments responsible for floral UV absorption, such as flavonoids, are also known for their protective effect against attack by herbivores [36,37,38] and photo damage caused by UV radiation [39,40,41]. Previous studies have shown that abiotic stress can induce the synthesis of such pigments [42,43,44] and that both intra- and inter-specific variations of UV reflectance in flowers correlate with geographic gradients of UV radiation [45,46,47,48]. Our results revealed a geographic cline of floral UV reflection in *P. rhoeas* that also correlates with a gradient of higher UV radiation toward the Mediterranean [49]. When red poppies are cultivated in Central Europe from seeds collected in the East Mediterranean, they develop petals with varying UV-reflecting patterns, i.e., petals with low UV reflectance, petals with substantial UV reflectance, and petals with intermediate patterns consisting of areas with low and substantial UV reflectance [35]. This variability indicates that in native populations of *P. rhoeas*, the synthesis of UV-absorbing pigments is influenced by environmental factors and suggests that its spread from the East Mediterranean westward into Europe could have triggered the occurrence of flower-color-polymorphic populations such as the ones observed in the North Mediterranean. Considering that along its distribution range *P. rhoeas* experiences very different environmental conditions, further investigations are needed to understand how biotic and abiotic factors might have influenced the color of *P. rhoeas* flowers. Despite the remaining questions about the mechanisms responsible for its color differences, we hypothesize that UV reflectance drove a shift in the pollination system of *P. rhoeas* that facilitated its spread across Europe.

Animal pollinators have long been considered to play a major role in the diversification of flowering plants [50,51,52,53]. It has been proposed that, through their selective behaviors, pollinators could mediate processes of floral isolation (“ethological isolation”, sensu Grant 1949), contributing to the origin and maintenance of ethological reproductive barriers [52,53,54]. Under such a scenario, diversification of floral characters would occur through initially polymorphic populations, consisting of variants for floral attributes promoting pollinator-mediated assortative mating [53]. The fact that Mediterranean red poppies develop petals with varying UV-reflecting patterns suggests that Central European populations of this species were initially composed of color-polymorphic variants with the potential of inducing selective behaviors of pollinators. Previous studies have shown that bees prefer red targets that reflect UV over pure red ones [55,56] and that patterns of floral UV reflectance increase the attraction of insect pollinators [57]. It can thus be proposed that in the absence of glaphyrid beetles, bees encountering color-polymorphic populations of *P. rhoeas* would favor the reproduction of individuals with UV-reflecting flowers. Alternatively, a small number of UV-reflecting flowering individuals could have given rise to Central European populations of *P. rhoeas* capable of attracting a wide variety of insect pollinators.

The red poppy is a common weed widely found in agricultural fields and perturbated sites throughout many temperate countries of the world [58]. Glaphyrid beetles from the genera *Eulasia* and *Pygopleurus* are the main pollinators of *P. rhoeas* along its native range [14,20,23,59]. The East Mediterranean is also the region of the highest diversity of these glaphyrid genera [60], which present sensory and morphological adaptations allowing them to specialize on red bowl-shaped flowers as feeding substrates [20,60]. As a self-incompatible species [17,18,19], the capacity of *P. rhoeas* to attract novel types of pollinators represents an essential process for its successful establishment outside its native range. The variability in flower color observed in Mediterranean *P. rhoeas* together with the evidence showing that floral pigments can be subject to modifications in response to different sources of stress [42,43,44] indicates that both biotic and abiotic factors need to be taken into account to understand the spread of the red poppy throughout different regions of the world.

The influence of human activities on the evolution of other species has been well-documented in plants with which humans have maintained long-lasting associations, such as crops and weeds [61,62,63,64]. Our study shows that such evolutionary changes can remain long un-noticed even in the case of a plant that has attracted our attention throughout history. Dispersal to new habitats can expose plants to drastic changes in pollinator environments, providing the opportunity to evaluate the impact of such perturbations over defined spatial, ecological, and temporal scales. Despite the need of further investigations to understand how pollinators contributed to the diversification of flower color in *P. rhoeas*, our work highlights introduced plants as useful models to study the role of pollinators in the evolution of angiosperms. Our results demonstrate that evolution of flower attractive traits can drive shifts in pollination systems that can promote the invasion of new environments by plants.

## 4. Materials and Methods

### 4.1. Plant Material

**Figure 5:**
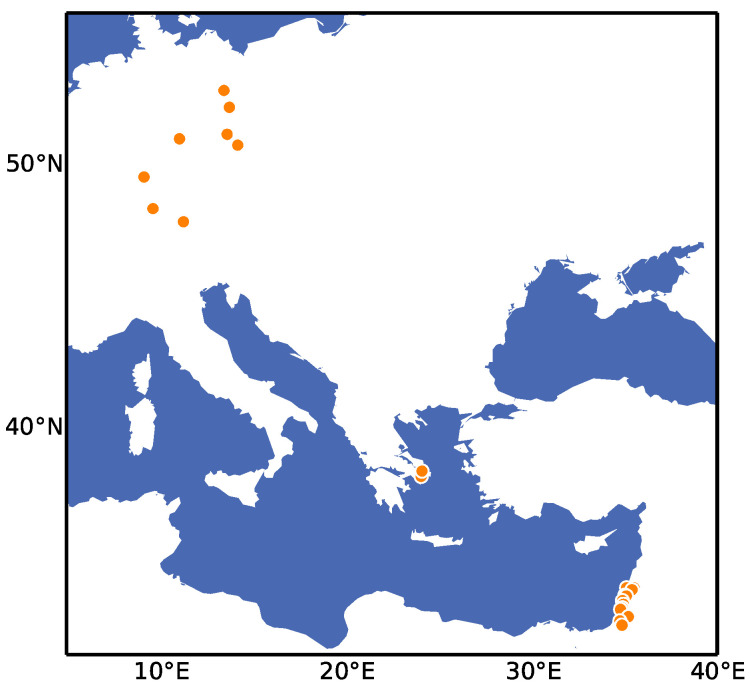
A map of the sites where flowers of *P. rhoeas* were collected for our study. Sites include ten locations in the East Mediterranean (Israel), two locations in the North Mediterranean (Greece), and eight locations in Central Europe (Germany).

Plant material was collected in the field and kept fresh until measurements were made. The colors of *P. rhoeas* flowers were studied at the peak of their flowering season at 10 locations in the Eastern Mediterranean (Israel), at 2 locations in the Northern Mediterranean (Greece), and at 8 different locations in Central Europe (Germany) (Figure 5).

### 4.2. Photography

Images of flowers were taken using a digital camera modified for increased UV sensitivity (EOS 10D, Canon USA Inc., Lake Success, NY, USA) with a quartz lens (105 mm, UV-Nikkor, Nikon, Tokyo, Japan). For UV exposures, a narrow bandpass filter was used (Baader Venus U-Filter, Baader Planetarium, Mammendorf, Germany) that consisted of a Schott UG11 substrate with dielectric coating that totally blocked wavelengths in the visible and infrared ranges while transmitting between 320 and 380 nm with a half-band width of 60 nm. For exposures in the visible spectrum, we used a broad bandpass filter that transmitted light between 400 and 700 nm.

### 4.3. Spectral Reflectance Measurements

The reflectance spectra of flowers and foliage collected in the field were measured between 300 and 700 nm using a spectrometer (SD2000; Ocean Optics, Dunedin, FL, USA). A white, diffuse reflectance tile was used as a reference for the measurements. Patches of petals were illuminated by a xenon lamp through an optic fiber while a second optic fiber collected the light reflected by the petals. The sample was illuminated under an angle of 45° to the optical axis of the fiber collecting the reflected light.

### 4.4. Modeling Insect Color Perception

Flower colors were plotted in two chromaticity diagrams using the receptor noise-limited (RNL) color-opponent model and the Maxwell triangle. According to the RNL model, colors can be depicted as points in a chromaticity diagram, where the discriminability between colors is given by the Euclidean distance between points (Figure 4c,d). The greater the distance, the more reliable is the discrimination [65,66]. This model accurately describes color discrimination in the honeybee [65,66] and in a number of other animals [67,68,69], indicating its value for psychophysical estimates of color discrimination. When *I*(*λ*) is the illumination spectrum for a flower or leaf with reflectance spectrum *S*(*λ*) (Figure 3), the quantum catch, Q*_k_*, of the short- (S), middle- (M), and long-wavelength (L)-sensitive photoreceptor *k* (*k* = S, M, L) with spectral sensitivity *R_k_*(*λ*) (Figure 4a,b) is

(1) $$Q_{k}=\int I\left(\lambda \right)S\left(\lambda \right)R_{k}\left(\lambda \right)d\lambda$$

A coordinate system (*q_k_*) was used where quantum catches for stimuli were divided by those for a reference stimulus or background, $Q_{k}^{b}$, to give a receptor contrast space.

(2) $$q_{k}=Q_{k}/Q_{k}^{b}$$

Assuming validity of Weber’s law, the color loci can be plotted with the coordinates

(3) $$X_{1}=A\left(f_{L}-f_{M}\right)$$

(4) $$X_{2}=B[f_{S}-\left(af_{L}+bf_{M}\right)]$$

where the receptor signals *f_k_* are

*f_k_* = ln(*q_k_*) (5)

with

(6) $$A=1/\sqrt{\omega_{L}^{2}+\omega_{M}^{2}}$$

(7) $$B=\sqrt{\left(\omega_{L}^{2}+\omega_{M}^{2}\right)/(\omega_{L}^{2}\omega_{M}^{2}+\omega_{S}^{2}\omega_{L}^{2}+\omega_{S}^{2}\omega_{M}^{2})}$$

(8) $$a=\omega_{M}^{2}/\left(\omega_{L}^{2}+\omega_{M}^{2}\right)$$

(9) $$b=\omega_{L}^{2}/\left(\omega_{L}^{2}+\omega_{M}^{2}\right)$$

Note that the axes do not correspond to opponent mechanisms. The Weber fractions or noise values, *ω_k_*, were set to *ω_S_* = 0.13, *ω_M_* = 0.06, and *ω_L_* = 0.12 [66]. The distance between the color loci can be expressed as

(10) $$\Delta S^{2}=\Delta X_{1}^{2}+\Delta X_{2}^{2}$$

The Maxwell triangle, on the other hand, does not make assumptions about the noise level of photoreceptor mechanisms, representing the most commonly used diagram in studies of trichromatic animals. According to this diagram, receptor coordinates are given by

(11) $$q_{k}^{c}=Q_{k}/\left(Q_{S}+Q_{M}+Q_{L}\right)$$

The location of a point, $q_{k}^{c}$, in the diagram determines the chromaticity of the color, and the length of the vector characterizes its luminosity or brightness [70]. To plot a point in the plane of the Maxwell triangle, Cartesian axes were used:

(12) $$X_{1}=\left(q_{L}^{c}-q_{M}^{c}\right)/\surd 2$$

(13) $$X_{2}=\surd \left(2/3\right)-\left[q_{S}^{c}-\left(q_{L}^{c}-q_{M}^{c}\right)/2\right]$$

Note that these axes are not related to opponent mechanisms. The vertices of the triangle are given by the following coordinates:

(14) S:[0,√(2/3)]

(15) M:[−1/√2,−1/√6]

(16) L:[1/√2,−1/√6]

## References

[1] Parker I.M. (1997). Pollinator limitation of *Cytisus scoparius* (Scotch broom), an invasive exotic shrub. Ecology.

[2] Larson K.C., Fowler S.P., Walker J.C. (2002). Lack of pollinators limits fruit set in the exotic *Lonicera japonica*. Am. Midl. Nat..

[3] Baker H.G. (1974). The evolution of weeds. Annu. Rev. Ecol. Syst..

[4] Lloyd D.G. (1992). Self- and cross-fertilization in plants. II. The selection of self- fertilization. Int. J. Plant. Sci..

[5] Morgan M.T., Wilson W.G. (2005). Self-fertilization and the escape from pollen limitation in variable pollination environments. Evolution.

[6] Eckert C.G., Kalisz S., Geber M.A., Sargent R., Elle E., Cheptou P.O., Goodwillie C., Johnston M.O., Kelly J.K., Moeller D.A. (2010). Plant mating systems in a changing world. Trends Ecol. Evol..

[7] Gabra S. (1954). *Papaver* species and opium through the ages. Bull. Inst. Egypte.

[8] Kadereit J.W. (1990). Some suggestions on the geographical origin of the central, west and north European synanthropic species of *Papaver* L.. Bot. J. Linn. Soc..

[9] Colledge S., Conolly J., Shennan S. (2004). Archaeobotanical Evidence for the Spread of Farming in the Eastern Mediterranean. Curr. Anthropol..

[10] Willerding U. (1986). Zur Geschichte der Unkräuter Mitteleuropas.

[11] Zohary D., Hopf M. (1993). Domestication of Plants in the Old World: The Origin and Spread of Cultivated Plants in West Asia, Europe, and the Nile Valley.

[12] Godwin H. (1975). The History of the British Flora: A Factual Basis for Phytogeography.

[13] McNaughton I.H., Harper J.L. (1960). The comparative biology of closely related species living in the same area I. External breeding barriers between *Papaver* species. New Phytol..

[14] Dafni A., Bernhardt P., Shmida A., Ivri Y., Greenbaum S. (1990). Red bowl-shaped flowers: Convergence for beetle pollination in the Mediterranean region. Isr. J. Bot..

[15] Dobson H.E.M., Groth I., Bergström G. (1996). Pollen advertisement: Chemical contrasts between whole-flower and pollen odors. Am. J. Bot..

[16] Proctor M., Yeo P. (1973). The Pollination of Flowers.

[17] Lawrence M.J. (1975). Genetics of self-incompatibility in *Papaver rhoeas*. Proc. R. Soc. B.

[18] Foote H.C., Ride J.P., Franklin-Tong V.E., Walker E.A., Lawrence M.J., Franklin F.C. (1994). Cloning and expression of a distinctive class of self-incompatibility (S) gene from *Papaver rhoeas* L.. Proc. Natl. Acad. Sci. USA.

[19] Wheeler M.J., de Graaf B.H.J., Hadjiosif N., Perry R.M., Poulter N.S., Osman K., Vatovec S., Harper A., Franklin F.C.H., Franklin-Tong V.E. (2009). Identification of the pollen self-incompatibility determinant in *Papaver rhoeas*. Nature.

[20] Martínez-Harms J., Vorobyev M., Schorn J., Shmida A., Keasar T., Homberg U., Schmeling F., Menzel R. (2012). Evidence of red sensitive photoreceptors in *Pygopleurus israelitus* (Glaphyridae: Coleoptera) and its implications for beetle pollination in the southeast Mediterranean. J. Comp. Physiol. A.

[21] Peitsch D., Fietz A., Hertel H., Desouza J., Ventura D.F., Menzel R. (1992). The spectral input systems of hymenopteran insects and their receptor-based color vision. J. Comp. Physiol. A.

[22] Martínez-Harms J., Palacios A.G., Márquez N., Estay P., Arroyo M.T.K., Mpodozis J. (2010). Can red flowers be conspicuous to bees? *Bombus dahlbomii* and South American temperate forest flowers as a case in point. J. Exp. Biol..

[23] Streinzer M., Roth N., Paulus H.F., Spaethe J. (2019). Color preference and spatial distribution of glaphyrid beetles suggest a key role in the maintenance of the color polymorphism in the peacock anemone (*Anemone pavonina*, Ranunculaceae) in Northern Greece. J. Comp. Physiol. A.

[24] Lotmar R. (1933). Neue Untersuchungen über den Farbensinn der Bienen, mit besonderer Berücksichtigung des Ultravioletts. Z. Vergl. Physiol..

[25] Kugler H. (1947). Hummeln und die UV-Reflexion an Kronblättern. Naturwissenschaften.

[26] Daumer K. (1958). Blumenfarben, wie sie die Bienen sehen. Z. Vergl. Physiol..

[27] Lunau K. (1993). Interspecific diversity and uniformity of flower colour patterns as cues for learned discrimination and innate detection of flowers. Experientia.

[28] Van der Kooi C.J., Stavenga D.G. (2019). Vividly coloured poppy flowers due to dense pigmentation and strong scattering in thin petals. J. Comp. Physiol. A.

[29] Chittka L., Waser N.M. (1997). Why red flowers are not invisible to bees. Isr. J. Plant. Sci..

[30] Sakai A.K., Allendorf F.W., Holt J.S., Lodge D.M., Molofsky J., With K.A., Baughman S., Cabin R.J., Cohen J.E., Ellstrand N.C. (2001). The population biology of invasive species. Annu. Rev. Ecol. Syst..

[31] Stockwell C.A., Hendry A.P., Kinnison M.T. (2003). Contemporary evolution meets conservation biology. Trends Ecol. Evol..

[32] Lambrinos J.G. (2004). How interactions between ecology and evolution influence contemporary invasion dynamics. Ecology.

[33] Barrett S.C.H., Colautti R.I., Eckert C.G. (2008). Plant reproductive systems and evolution during biological invasion. Mol. Ecol..

[34] Richardson D.M., Allsopp N., D’Antonio C.M., Milton S.J., Rejmanek M. (2000). Plant invasions—The role of mutualisms. Biol. Rev..

[35] Dudek B., Schneider B., Hartmut H.H., Stavenga D.G., Martínez-Harms J. (2020). Highly different flavonol content explains geographic variations in the UV reflecting properties of flowers of the corn poppy, *Papaver rhoeas* (*Papaveraceae*). Phytochemistry.

[36] Haribal M.M., Feeny P.P. (2003). Combined roles of contact stimulant and deterrents in assessment of host-plant quality by ovipositing zebra swallowtail butterflies. J. Chem. Ecol..

[37] Mallikarjuna N., Kranthi K.R., Jadhav D.R., Kranthi S., Chandra S. (2004). Influence of foliar chemical compounds on the development of *Spodoptera litura* (Fab.) in interspecific derivatives of groundnut. J. Appl. Entomol..

[38] Sosa T., Chaves N., Alias J.C., Escudero J.C., Henao F., Gutiérrez-Merino C. (2004). Inhibition of mouth skeletal muscle relaxation by flavonoids of *Cistus ladanifer* L.: A plant defense mechanism against herbivores. J. Chem. Ecol..

[39] Hollósy F. (2002). Effects of ultraviolet radiation on plant cells. Micron.

[40] Rozema J., van de Staaij J., Björn L.O., Caldwell M. (1997). UV-B as an environmental factor in plant life: Stress and regulation. Trends Ecol. Evol..

[41] Shirley B.W. (1996). Flavonoid biosynthesis: ‘new’ functions for an ‘old’ pathway. Trends Plant. Sci..

[42] Havaux M., Kloppstech K. (2001). The protective functions of carotenoid and flavonoid pigments against excess visible radiation at chilling temperature investigated in *Arabidopsis npq* and *tt* mutants. Planta.

[43] Albert A., Sareedenchai V., Heller W., Seidlitz H.K., Zidorn C. (2009). Temperature is the key to altitudinal variation of phenolics in *Arnica montana* L. cv. ARBO. Oecologia.

[44] Jansen M.A.K., Gaba V., Greenberg B.M. (1998). Higher plants and UV-B radiation: Balancing damage, repair and acclimation. Trends Plant. Sci..

[45] Del Valle J.C., Buide M.L., Casimiro-Soriguer I., Whittall J.B., Narbona E. (2015). On flavonoid accumulation in different plant parts: Variation patterns among individuals and populations in the shore campion (*Silene littorea*). Front. Plant. Sci..

[46] Koski M.H., Ashman T.-L. (2015). An altitudinal cline in UV floral pattern corresponds with a behavioral change of a generalist pollinator assemblage. Ecology.

[47] Koski M.H., Ashman T.-L. (2015). Floral pigmentation patterns provide an example of Gloger’s rule in plants. Nat. Plants.

[48] Jaakola L., Hohtola A. (2010). Effect of latitude on flavonoid biosynthesis in plants. Plant. Cell Env..

[49] Seckmeyer G., Pissulla D., Glandorf M., Henriques D., Johnsen B., Webb A., Siani A.-M., Bais A., Kjeldstad B., Brogniez C. (2008). Variability of UV irradiance in Europe. Photochem. Photobiol..

[50] Darwin C. (1876). The Effects of Cross and Self Fertilization in the Vegetable Kingdom.

[51] Grant V. (1949). Pollination systems as isolating mechanisms in angiosperms. Evolution.

[52] Levin D.A. (1971). The origin of reproductive isolating mechanisms in flowering plants. Taxon.

[53] Grant V. (1994). Modes and origins of mechanical and ethological isolation in angiosperms. Proc. Natl. Acad. Sci. USA.

[54] Grant V. (1952). Isolation and hybridization between *Aquilegia formosa* and *A. pubescens*. El Aliso.

[55] Chen Z., Liu C.-Q., Sun H., Niu Y. (2020). The ultraviolet colour component enhances the attractiveness of red flowers of a bee-pollinated plant. J. Plant. Ecol..

[56] Lunau K., Papiorek S., Eltz T., Sazima M. (2011). Avoidance of achromatic colours by bees provides a private niche for hummingbirds. J. Exp. Biol..

[57] Koski M.H., Ashman T.-L. (2014). Dissecting pollinator responses to a ubiquitous ultraviolet floral pattern in the wild. Funct. Ecol..

[58] Mitich L.W. (2000). Corn Poppy (*Papaver rhoeas* L.). Weed Technol..

[59] Keasar T., Harari A.R., Sabatinelli G., Keith D., Dafni A., Shavit O., Zylbertal A., Shmida A. (2010). Red anemone guild flowers as focal places for mating and feeding by Levant glaphyrid beetles. Bot. J. Linn. Soc..

[60] Sabatinelli G., Eberle J., Fabrizi S., Ahrens D. (2020). A molecular phylogeny of Glaphyridae (Coleoptera: Scarabaeoidea): Evolution of pollination and association with ‘Poppy guild’ flowers. Syst. Entomol..

[61] Meyer R.S., Purugganan M.D. (2013). Evolution of crop species: Genetics of domestication and diversification. Nat. Rev. Genet..

[62] Darwin C. (1868). The Variation of Animals and Plants under Domestication.

[63] Dewet J.M.J., Harlan J.R. (1975). Weeds and domesticates: Evolution in man-made habitat. Econ. Bot..

[64] Barrett S.H. (1983). Crop mimicry in weeds. Econ. Bot..

[65] Vorobyev M., Osorio D. (1998). Receptor noise as a determinant of colour thresholds. Proc. R. Soc. B.

[66] Vorobyev M., Brandt R., Peitsch D., Laughlin S.B., Menzel R. (2001). Colour thresholds and receptor noise: Behaviour and physiology compared. Vis. Res..

[67] Koshitaka H., Kinoshita M., Vorobyev M., Arikawa K. (2008). Tetrachromacy in a butterfly that has eight varieties of spectral receptors. Proc. R. Soc. B.

[68] Goldsmith T.H., Butler B.K. (2003). The roles of receptor noise and cone oil droplets in the photopic spectral sensitivity of the budgerigar, *Melopsittacus undulatus*. J. Comp. Physiol. A.

[69] Lind O., Chavez J., Kelber A. (2013). The contribution of single and double cones to spectral sensitivity in budgerigars during changing light conditions. J. Comp. Physiol. A.

[70] Wyszecki G., Stiles W.S. (1982). Color Science: Concepts and Methods, Quantitative Data and Formulae.

